# Plasma metabolomics reveals distinct responses to acute and chronic heat stress in broilers

**DOI:** 10.1016/j.psj.2026.106876

**Published:** 2026-03-28

**Authors:** A. Papanikolaou, E.M.J.M. Kampschoer, M.G.R. Matthijs, J.T. Schonewille, S. Khempaka, C.R. Berkers, E.A. Zaal

**Affiliations:** a Division of Farm Animal Health, Department of Population Health Sciences, Faculty of Veterinary Medicine, Utrecht University, Yalelaan 7, 3584 CM Utrecht, Netherlands; b School of Animal Technology and Innovation, Institute of Agricultural Technology, Suranaree University of Technology, Nakhon Ratchasima 30000, Thailand; c Division of Cell Biology, Metabolism and Cancer, Department of Biomolecular Health Sciences, Faculty of Veterinary Medicine, Utrecht University, Yalelaan 2, 3584 CM Utrecht, Netherlands

**Keywords:** Broilers, Heat stress, Metabolomics, Plasma, Amino acid metabolism

## Abstract

Heat stress (HS) poses a significant threat to the global broiler industry by impairing growth performance and welfare of the birds. The metabolic adaptations to varying durations of heat exposure remain poorly understood. This study aimed to characterize the plasma metabolic profiles of broilers subjected to thermoneutral conditions (TN; 32-22°C, age depended), very short-term (8 h), intermediate (1 week), and long-term (5 weeks) cyclic heat stress (HS; 31°C on average), as well as a subsequent recovery (REC) phase after a 1-week period of HS. A total of 450 day-old male Cobb 500 broilers were randomly allocated to five treatments. Each treatment consisted of 6 replicate pens, with 15 birds per pen. The treatments were distributed in two environmentally controlled rooms based on their assigned climatic conditions (thermoneutral or heat stress). We evaluated growth performance, cloaca temperature, and performed liquid chromatography-mass spectrometry (LC-MS) based metabolomics on plasma from six broilers per treatment. As expected, exposure to heat stress for 1 week or longer significantly increased cloaca temperature (>1.5°C), reduced feed intake, and impaired growth performance. Metabolomic analysis revealed distinct, duration-dependent metabolic signatures. Very short-term (8 h) heat stress was uniquely characterized by elevated plasma uridine, suggesting an activation of nucleotide salvage pathways. In contrast, long-term (5 weeks) heat stress induced a catabolic phenotype, evidenced by increased plasma levels of kynurenine, creatinine, and argininosuccinate, reflecting muscle proteolysis and altered amino acid metabolism. Both short- and long-term heat stress suppressed de novo nucleotide synthesis, marked by decreased orotate and N-carbamoyl-aspartate. Notably, these metabolic alterations were largely reversible, with the profiles of recovery broilers returning to thermoneutral levels within 8 hours. Our findings demonstrate that the broilers’ metabolic response to heat stress is a dynamic, duration-dependent process. The identified metabolites, particularly uridine and kynurenine, represent promising candidate biomarkers for distinguishing between acute and chronic heat stress, offering a valuable tool for improving welfare monitoring in poultry production.

## Introduction

The combination of increasing occurrence of heat waves all over the world and the increasing broiler production in (sub)tropical regions, has resulted in concerns regarding heat adaptation of broilers, globally. Broilers are bred and raised for their fast growth rate, which is accompanied by elevated metabolic heat production, rendering them less suitable for production systems in high temperature climates and in temperate climates with recurring heat waves. As previously reported, a high environmental temperature induces heat stress, resulting in lower growth rate, lower feed efficiency, and in extreme cases, higher mortality (Liu et al., 2020; Mangan and Siwek, 2024; Nawab et al., 2018). Furthermore, both prolonged consecutive exposure and extended daily exposure to high temperatures exacerbate consequences for broilers (Teyssier et al., 2022).

The use of metabolomics for the identification of the metabolic profile of livestock in various conditions has been used before in pigs but also in broilers (Cui et al., 2019; Lu et al., 2018; Tomonaga et al., 2018). Metabolomics, together with other -omics techniques, enables comprehensive insights into the adaptation of animals to external conditions, such as heat stress. Through metabolomic profiling of blood plasma, the identification of metabolites and associated novel pathways can offer new insights into the mechanism underlying heat stress. The acquired knowledge enables targeted research into potential intervention strategies that can be applied in practice to mitigate the negative effects of heat stress in broilers.

While previous metabolomic studies have investigated heat stress in broilers, the duration of exposure has received little attention (Zampiga et al., 2021). According to Xie et al. (2015), the metabolic response to heat stress is strictly duration-dependent in feed-restricted broiler breeders. Short-term, acute exposure elicits rapid physiological defence mechanisms to maintain homeostasis, whereas prolonged chronic exposure induces systemic adaptations and eventual tissue damage as the birds attempt to acclimate. Although these distinct phases have been described, a continuous characterization of the plasma metabolome across varying exposure durations in *ad libitum* fed broilers remains lacking. Therefore, the primary aim of this study was to characterize the plasma metabolic profile in of broilers under three distinct conditions: thermoneutral, very short-term (8 h), and long-term (5 weeks) cyclic heat stress. After identifying the adapting metabolic pathways in these conditions, a secondary aim of this study was to evaluate the same pathways in broilers exposed to short-term (1 week) heat stress and in a recovery treatment with conditions returning to thermoneutral after 1 week of heat stress. Profiling these duration-dependent metabolic shifts not only provides a potential biomarker tool for monitoring heat stress, but also serves as a baseline to evaluate the efficacy of future mitigating interventions.

## Material and methods

### Ethical considerations

The current experiment was approved by the Animal Ethics Committee of Suranaree University of Technology Isan (SUT, Approval number: SUT-IACUC-008/2023) and based on the Ethics of Animal Experimentation of the National Research Council of Thailand.

### Animals, housing, diets and experimental design

The experiment was conducted at the facilities of Suranaree University of Technology during the months April and May during which the climate is tropical. Four hundred fifty (450) Cobb500 slow feather broilers were used for this study. Broilers were obtained from a commercial hatchery (Chonburi, Thailand) and were acquired as male broilers, based on feather-sexing. To ensure uniform starting conditions, the broilers were distributed into pens using a stratified random design based on body weight. Animals were weighed upon arrival, classified into specific weight categories, and allocated such that every pen received an equal distribution of weight classes, ensuring identical cumulative starting weights across all experimental treatments. In total, 5 treatments were included in the parallel experimental design, with 6 replicates per treatment and 15 broilers per replicate, housed in one pen (Table 1). Two broiler houses were used for the different climatic conditions: one air-conditioned, climate controlled, broiler house (**CC**), mimicking thermoneutral conditions and one open sided broiler house (**OS**) (Table 1). The total experimental period lasted 36 days. This duration was chosen to evaluate the acute heat stress and recovery responses without the risk of excessive mortality seen in heavier broilers. Broilers of treatment 1 were raised according to the Cobb500 management guide throughout the experimental period of 5 weeks (days 1 to 36) in the CC broiler house, mimicking thermoneutral conditions (**TN-5wk**) (Table 1). Broilers of treatment 2 were raised identical to treatment 1 for 5 weeks before being placed into an OS broiler house for 8 hours before sampling on day 36 (**HS-8h**). Broilers of treatment 3 were raised in the open sided broiler house the whole experimental period of 5 weeks (days 1 to 36) (**HS-5wk**). Broilers of treatment 4 were raised in the CC house for 4 weeks (days 1 to 28) and then placed into the OS broiler house for the remaining week (days 29 to 36) (**HS-1wk**). Treatment 5 was a separate, recovery treatment; the broilers were housed in the CC broiler house for 4 weeks (days 1 to 28), then transferred to the OS house for 1 week (days 29 to 35), and finally transferred back to the CC house for the last 8 hours of the experimental period on day 36 (**REC**).

**Table 1:** Overview of the experimental treatments. The broilers were either housed in a climate-controlled house (CC) or in an open-sided house (OS) where broilers were subjected to the prevailing environmental climate.

	Experimental treatments^1^										
Days during experiment	Cobb500 guideline^4^	TN-5wk		HS-8h		HS-5wk		HS-1wk		REC	
		House	°C^2^	House	°C	House	°C	House	°C	House	°C
Day 1 to 7	30.1	CC	32.3 ± 2.7	CC	32.3 ± 2.6	OS	32.7 ± 3.7	CC	32.7 ± 2.8	CC	32.4 ± 2.7
Day 8 to 14	27.9	CC	27.1 ± 0.9	CC	26.5 ± 1.0	OS	31.1 ± 4.8	CC	25.5 ± 1.2	CC	26.8 ± 0.9
Day 15 to 21	25.7	CC	26.2 ± 1.2	CC	24.8 ± 1.4	OS	31.5 ± 4.5	CC	25.8 ± 1.4	CC	26.5 ± 1.4
Day 22 to 28	23.5	CC	23.9 ± 2.1	CC	22.8 ± 2.1	OS	31.9 ± 3.9	CC	23.4 ± 2.6	CC	23.7 ± 2.2
Day 29 to 35	21.7	CC	22.3 ± 2.5	CC	22.4 ± 4.6	OS	32.8 ± 3.9	OS	31.9 ± 4.8	OS	30.9 + 5.4
Day 36^3^	20.0	CC	20.6 ± 1.4	OS	37.4 ± 1.2	OS	37.6 ± 1.2	OS	37.3 ± 1.1	CC	20.5 ± 1.7

1 TN = Thermoneutral, HS = Heat stress. The treatments were also described as; control (TN-5wk), very short-term heat stress (HS-8 h), long term heat stress (HS-5wk), short term heat stress (HS-1wk) and recovery (REC).

2 Except for day 36, the temperature represents the average, 24 h temperature measured (Tempmate S2 loggers, Tempmate GmbH, Germany) during the week in question.

3 The experiment ended on day 36 at 18:00. The reallocation of birds, from CC to OS (HS-8 h) or vice versa (REC) occurred at 10:00. The temperatures indicated on day 36 represent the 8 h period.

4 The temperature recommendations are according to the Cobb500 management guide and were adapted based on the stocking density of the pens.

All transfers of broilers were done on the early morning of the following week on day, 29 (HS-1wk, REC), 36 (HS-8 h, REC). Samples were collected in the afternoon of day 36, just before the end of the experiment.

All broilers were housed on concrete floors covered with rice husks (7.4 kg/m^2^), with pen dimensions of 1.12 × 0.82 meters. The area was calculated according to the expected final body weight and maximum stocking density (42 kg/m^2^, according to EU Council Directive 2007/43/EC).

The ingredient- and nutrient composition of the diets was identical across the treatments and consisted of a starter, grower and finisher diet (Table 2). The starter diet was provided during the first 12 days of age, subsequently followed by the grower diet (13-28 days) and the finisher diet (29-36 days). All diets were formulated to meet or exceed the nutrient requirements as provided by the National Research Council & Subcommittee on Poultry Nutrition (1994) and the Cobb500 broiler nutritional recommendations. Diets were provided in mash form using tower feeders (⌀ 42 cm). Water was provided ad libitum in bell drinkers (⌀ 30 cm) and was replenished twice daily at 08:00 and 16:00.

**Table 2:** Composition of the diet during the starter and grower periods.

Composition (%)	Starter (1-12 d)	Grower 1 (13-28 d)	Grower 2 (29-36 d)
Corn	56.48	63.13	64.43
Soybean meal (44 % CP)	36.20	31.00	28.85
Palm oil	1.80	1.12	2.41
Calcium carbonate, CaCO_3_	1.23	1.23	1.15
Monocalcium phosphate, Ca(H_2_PO_4_)_2_	2.10	1.27	1.15
Salt	0.52	0.52	0.52
Premix^1^	0.50	0.50	0.50
L-lysine HCl 79 %	0.34	0.35	0.29
DL-methionine	0.39	0.37	0.32
L-threonine	0.21	0.18	0.13
L-Arginine	0.14	0.16	0.13
L-isoleucine	0.04	0.06	0.03
L-valine	0.05	0.11	0.09
Calculated composition (%)			
Metabolizable energy (kcal/kg)	2900	2950	3050
Crude protein	21.51	19.75	18.66
Crude fat	3.96	3.47	4.78
Crude fibre	3.82	3.60	3.48
Calcium	0.96	0.80	0.74
Phosphorus	0.83	0.64	0.61
Available phosphorus	0.58	0.40	0.37
Digestible Lys	1.26	1.16	1.06
Digestible Met	0.67	0.63	0.57
Digestible Met + Cys	0.94	0.88	0.82
Digestible Thr	0.86	0.78	0.70
Digestible Arg	1.36	1.25	1.16
Digestible Ileu	0.81	0.75	0.69
Digestible Val	0.89	0.88	0.82
Digestible Tryp	0.26	0.23	0.22
Digestible His	0.49	0.45	0.43
Digestible Leu	1.59	1.49	1.44

1 Premix (0.5 %) provided the following (per kg of diet): vitamin A (trans-retinyl acetate) 15000 IU; vitamin D3 (cholcalciferol) 3000 IU; vitamin E (DL-a-tocopherol) 25 IU; vitamin K3 (menadione nicotinamide bisulphite) 5 mg; thiamine (thiamine-mononitrate) 2 mg; riboflavin 7 mg; pyridoxine (pyridoxine. HCl) 4 mg; vitamin B12 (cyanocobalamin) 25 µg; pantothenic acid (D-Ca pantothenate) 11.04 mg; niacin (nicotinic acid) 35 mg; folic acid 1 mg; biotin 15 µg; choline chloride 250 mg; Cu (copper sulfate) 6.4 mg; Mn (manganese oxide) 100 mg; Zn (zinc oxide) 75 mg; Fe (iron sulfate) 0.40 g; I (calcium iodate) 0.40 mg; Se (sodium selenite) 0.36 mg.

Brooding heat was provided throughout the first week after hatch in both houses using an infrared heat lamp bulb (175 W, one per pen), initially placed approximately 20 cm above the broilers. The temperature loggers were positioned outside the reach of the infrared bulbs, and therefore thermal comfort during the first week was managed by continuously monitoring chick distribution and behaviour, adjusting the lamp heights accordingly to prevent huddling or avoidance. The temperature and humidity of the broilers in the OS house were completely dependent on the ambient climate of the region after the first week, due to the natural ventilation of the open-sided structure. The region (approx. 14°52′N, 102°00′E) has a tropical climate in the particular season (March-April) when the experiment was performed. The climate conditions of the CC house were regulated through three air-conditioning units (York air conditioner, 40000 btu, York, USA), which kept the temperature in the required range for the particular age of the broilers. The temperature range was defined according to the Cobb500 Handbook (Cobb-Vantress, 2021) in combination with the expected stocking density per pen. The resulting recommended temperature range is shown in Table 1. In addition, humidity was regulated by an industrial dehumidifier (UDR-890, Unic Climate Professional & Engineering Co., Ltd. Thailand) and the intended relative humidity was 60 %. Temperature and relative humidity were measured continuously (with an interval of 10 minutes) with two Tempmate S2 loggers (Tempmate GmbH, Germany) per treatment, placed at broiler height.

### Environmental temperature and relative humidity

**Fig. 1:**
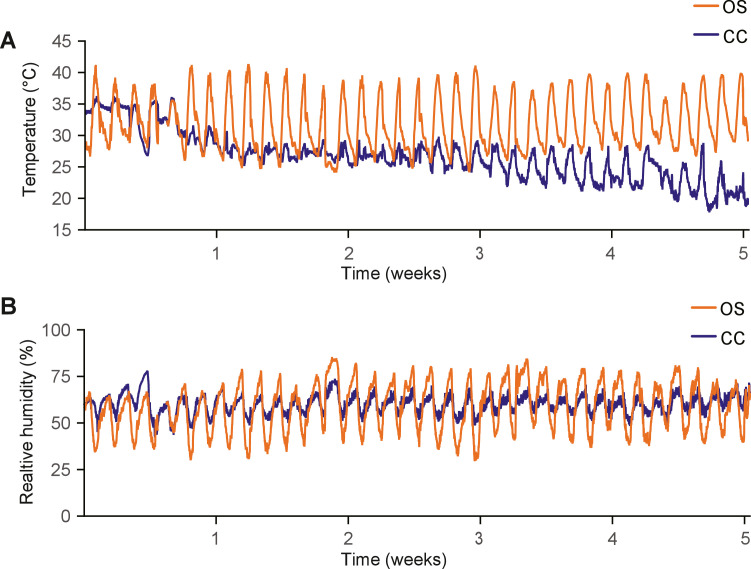
Environmental temperature and relative humidity during the experimental period in the two broiler houses. Blue and orange lines depict the temperature and relative humidity in the climate-controlled house and the open-sided house, respectively.

The continuous temperature and humidity readings for each broiler house are illustrated in Fig. 1A, with specific treatment averages detailed in Table 1. Briefly, to meet standard Cobb500 recommendations, the temperature in the CC house was gradually decreased from approximately 32°C to 23°C over the first 3 weeks, and subsequently maintained at an average of 22-23°C from day 22 to day 36. (Table 1). The temperature in the OS house remained on average 31°C throughout the experimental period (Table 1).

On day 36, the HS-8 h treatment group was exposed to an 8-hour heat stress challenge. During this period, the average temperature was 37.4°C (SD = 1.2). On the other hand, the REC treatment group was returned to TN conditions for the final 8 hours of the experiment, in which period the average temperature was 20.5°C (SD = 1.7).

Relative humidity in the CC house ranged between 48 and 78 %, whilst in the OS house the range varied from 36 to 88 % (Fig. 1B). On average the relative humidity in the CC house was 62 % (SD = 5 %) and 62 % (SD = 13 %) in OS house.

### Data and sample collection

Feed intake (**FI**) and bodyweight were recorded weekly in the morning of the first day of each week. After removing the tower feeders simultaneously from all pens, the leftover feed was weighed and subtracted from the offered feed. In the meantime, all broilers were weighed individually.

Cloaca temperature was measured using a digital thermometer (Digital Veterinary thermometer SC12, SCALA Electronic GmbH, Stahnsdorf, Germany) from one broiler per pen, with bodyweight closest to the average pen bodyweight. The measurements were conducted at 16:00 on the first day of each week.

Blood was collected from the same broilers in the afternoon (16.00) on the last day of the experimental period (day 36). The blood was sampled from the brachial vein (∼ 0.5 ml) into a lithium heparin coated tube within 30 seconds of measuring the cloaca temperature. A 24 Gauge (0.5 mm x 25 mm) needle (Sterican®, B. Braun, Melsungen, Germany) was used in combination with a 3 ml syringe (Sterican®, B. Braun, Melsungen, Germany). The blood samples were stored on ice for a maximum of 2 h and once all blood samples were collected, they were centrifuged (∼1,200 g) for 15 min, and the plasma was stored at −20°C until analysis. Two broilers from treatment HS-5wk died before sampling and therefore no blood was collected.

### Metabolomics analysis

Plasma samples were thawed on ice and 10 µl of plasma was mixed with 990 µl methanol/acetonitrile/dH_2_O (2:2:1) to extract metabolites. Samples were centrifuged at 16,000 g for 15 minutes at 4°C to remove cell debris and proteins. Supernatants were collected for Liquid Chromatography-Mass Spectrometry (LC-MS) analysis.

LC-MS analysis was performed on a Q-Exactive HF mass spectrometer (Thermo Scientific) coupled to a Vanquish autosampler and pump (Thermo Scientific). The MS operated in polarity-switching mode with spray voltages of 4.5 kV and −3.5 kV. Metabolites were separated on a Sequant ZIC-pHILIC column (2.1 × 150 mm, 5 μm, guard column 2.1 × 20 mm, 5 μm; Merck) with elution buffers acetonitrile and eluent A (20 mM (NH_4_)_2_CO_3_, 0.1 % NH_4_OH in ULC/MS grade water (Biosolve)). Gradient ran from 20 % eluent A to 60 % eluent A in 20 minutes, followed by a wash step at 80 % and equilibration at 20 %. Flow rate was set at 100 µl/min. Data analysis was performed using Tracefinder software (Thermo Scientific). Metabolites were identified and quantified on the basis of exact mass within 5 ppm and further validated by concordance with retention times of standards. Peak intensities were normalized based on total ion count.

### Statistical analysis

One- and two-way ANOVA with Tukey’s post hoc test was performed using GraphPad Prism (version 10.4.1 for Windows, GraphPad Software, Boston, Massachusetts USA, www.graphpad.com) on growth performance parameters and the cloaca temperature.

For metabolomics analysis, univariate analysis was performed using Student’s t-tests with Benjamini-Hochberg post hoc correction (R version 4.5.2, http://www.r-project.org), to compare metabolite levels between groups. For multivariate analysis, principal component analysis (PCA) and partial least squares-discriminant analysis (PLS-DA) was applied to identify metabolite groups contributing to group separation. Variable importance in projection (VIP) scores > 1 with a significant difference (*P* < 0.05, False Discovery Rate-adjusted) between at least one pair of experimental groups were considered to have high discriminatory power. Metabolite Set Enrichment Analysis (MSEA) was performed using MetaboAnalyst 6.0 (Pang et al., 2024).

## Results

### Cloaca temperature measurements

**Fig. 2:**
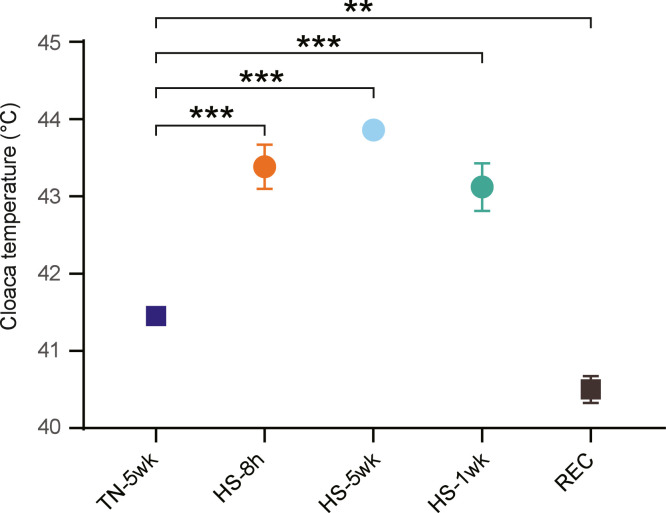
Cloaca temperature of broilers allocated to different treatments on the last day of the experimental period. Rectangles represent broilers located in a climate controlled (CC) barn during measuring, while circles represent broilers located in an open sided (OS) barn. P-values indicate statistical difference with TN-5wk broilers. Error bars depict the standard error of each treatment. TN-5wk: broilers housed in a climate-controlled house for 5 weeks, HS-8h: broilers housed in a climate-controlled broiler house for 5 weeks and transferred to an open-sided house for 8 h on the last day, HS-5wk: broilers housed in an open-sided broiler house for 5 weeks, HS-1wk: broilers housed in a climate-controlled broiler house for 4 weeks and transferred to an open sided house for the final week, REC: broilers housed in a climate-controlled broiler house for 3 weeks, transferred to the open-sided house for 1 week and back to the climate-controlled broiler house for the final week.

The average cloaca temperature increases at least 1.5°C in all broilers that were in the OS house during measuring, irrespective of age (Fig. 2) (*P* < 0.001). Broilers of TN-5wk had a lower cloaca temperature 41.4°C (SD = 0.23) compared to the HS treatments. In more detail, HS-8 h, HS-5wk and HS-1wk had a mean cloaca temperature of 43.4°C (SD = 0.70), 43.9°C (SD = 0.05) and 43.1°C (SD = 0.69), respectively. In contrast, the cloaca temperature of the broilers of the REC treatment was lower (*P* *=* 0.009) than the TN-5wk broilers at the end of week 5 (41.4°C, SD = 0.23), 8 hours after the transfer to the CC house, i.e., 40.5°C (SD = 0.42).

### Growth performance

At week 4 of the experimental period, the body weight of the TN-5wk broilers was 35 % higher than the HS-5wk broilers (*P* *<* 0.001) (Table 3). By week 5, the TN-5wk broilers’ body weight was 53 %, 24 % and 22 % higher than the final bodyweights of the HS-5wk, HS-1wk, and REC respectively (*P* *<* 0.001). However, the body weight of the HS-8 h broilers was similar to the TN-5wk broilers (*P* = 0.889).

**Table 3:** Growth performance of broilers allocated to rooms with different climatic conditions.

	TN-5wk	HS-8h	HS-5wk	HS-1wk	REC	SEM	*P*-value
Body weight^1^							
Day 8	196	191	183	190	190	0.90	>0.992
Day 29	1732^a^	1747^a^	1286^b^	1699^a^	1681^a^	7.47	<0.001
Day 36	2569^a^	2536^a^	1683^c^	2077^b^	2099^b^	12.69	<0.001
Feed intake (g/d)							
Days 29-36	203^a^	199^a^	138^b^	154^b^	145^b^	1.35	<0.001
Gain (g/d)							
Days 29-36	120^a^	113^a^	57^b^	54^b^	60^b^	0.05	<0.001
FCR							
Days 29-36	1.700^a^	1.770^a^	2.463^b^	2.928^b^	2.470^b^	0.03	<0.001

^a,b,c^Results without common superscript indicate statistical significant difference between treatments (*P* < 0.05). FCR: feed conversion ratio.

1 Initial body weight was similar (pooled SEM = 0.25, *P* > 0.999) between treatments and the mean value across all treatments was 43.3 g/broiler TN-5wk: Broilers housed under thermoneutral conditions for 5 weeks, HS-8h: Broilers exposed to heat stress for 8 hours, HS-5wk: broilers exposed to heat stress for 5 weeks, HS-1wk: Broilers exposed to heat stress for 1 week (HS-1wk), and REC: Broilers housed under thermoneutral conditions for 3 weeks, transferred to heat stress conditions for 1 week and back to the thermoneutral conditions for the final week.

Feed intake during week 5 in the TN-5wk and HS-8 h broilers was similar (*P* = 0.989), however, the feed intake of all other treatments was approx. 26 % lower (*P* < 0.001). The daily bodyweight gain was also higher in the TN-5wk and HS-8 h treatments (*P* = 0.676), and almost 50 % lower in the remaining treatments (*P* < 0.001).

The feed conversion ratio (FCR) followed the same pattern as feed intake and bodyweight gain during this period resulting in a higher FCR in HS-5wk, HS-1wk and REC compared to TN-5wk and HS-8 h treatments (*P* < 0.009).

### Metabolic responses to long (HS-5wk) and very short-term (HS-8 h) heat stress

**Fig. 3:**
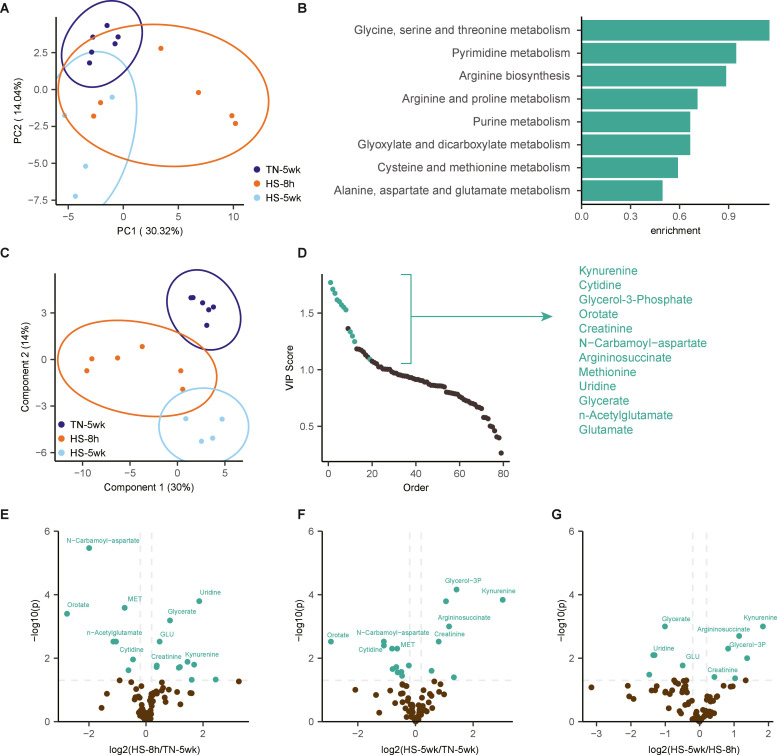
Serum metabolomics analysis of broilers exposed to long-term and very short-term heat stress. LC-MS based metabolomics was performed on serum of broilers housed under thermoneutral conditions for 5 weeks (TN-5wk), broilers kept under thermoneutral conditions for 5 weeks and subsequently transferred to an open-sided house for 8 hours (HS-8 h), and broilers housed in an open-sided broiler house for 5 weeks (HS-5wk). A: Principal component analysis (PCA) of serum metabolite levels from TN-5wk (dark blue), HS-8 h (orange) and HS-5wk (light blue). B: Pathway enrichment analysis of the top 20 metabolites contributing to separation along principal component 1. C: Partial least squares-discriminant analysis (PLS-DA) of serum metabolite levels from TN-5wk (dark blue), HS-8 h (orange) and HS-5wk (light blue). D: Variable importance in projection (VIP) scores of metabolites ranked by their contribution to group separation. Metabolites with a VIP score > 1 and a statistically significant difference between at least two treatments (*P* < 0.05) are highlighted in green and considered key contributors to the observed metabolic shifts. E-G: Volcano plots showing differential serum metabolites between (E) TN-5wk vs HS-8 h, (F) TN-5wk vs HS-5wk and (G) HS-5wk vs HS-8 h. Metabolites are plotted according to their statistical significance (y-axis) and fold change (x-axis). Colored points indicate significantly up- or downregulated metabolites. Metabolites that were identified as key regulators in Fig. D are annotated.

LC-MS based metabolomic profiling of plasma samples from TN-5wk, HS-8 h and HS-5wk broilers detected a total number of 79 metabolites across all samples. Multivariate analysis using PCA demonstrated a partial separation between treatments (Fig. 3A). While the TN-5wk and HS-5wk treatments were clearly separated along PC2, the HS-8 h broilers had some overlap with the HS-5wk broilers. These results indicate a global alteration of the plasma metabolome in response to both long-term (5 weeks) and very short-term (8 hours) heat stress.

To gain further insight into the metabolic pathways associated with the response of the HS-8 h broilers, we performed MSEA on the top 20 metabolites contributing to separation along PC1, where most samples of the HS-8 h treatment were distinguished from samples of HS-5wk and TN-5wk treatments. MSEA highlighted enrichment of pathways related to pyrimidine and purine metabolism and amino acid metabolism, for example arginine metabolism (Fig. 3B).

PLS-DA identified specific metabolites responsible for distinguishing the experimental treatments (Fig. 3C). Metabolites with both a variable importance in projection (VIP) score > 1 and a difference between at least two treatments (*P* *<* 0.05), were considered key contributors to the observed metabolic shifts (Fig. 3D-G). These metabolites included kynurenine, orotate, creatine, argininosuccinate, uridine and glutamate, which are involved in the pathways of amino acid metabolism and nucleotide turnover.

**Fig. 4:**
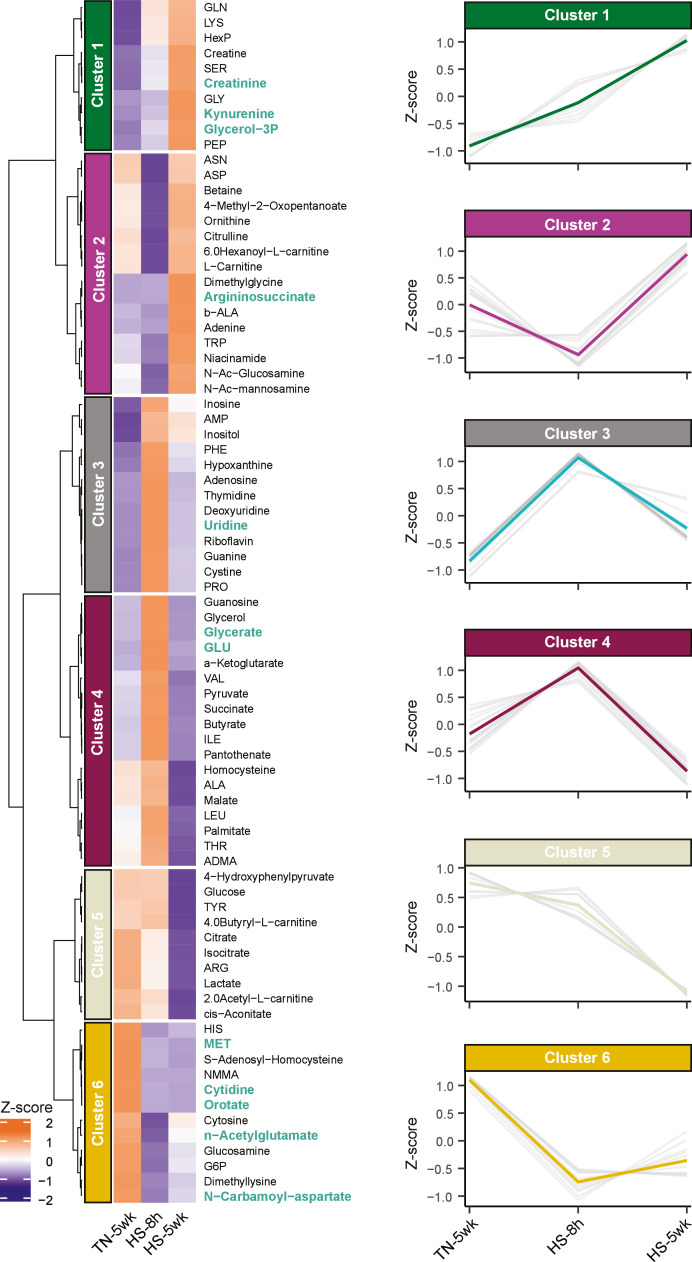
Hierarchal clustering of serum metabolites and cluster-specific metabolic profiles in broilers under heat stress. Hierarchical clustering analysis was performed on all detected serum metabolites from broilers housed under thermoneutral conditions for 5 weeks (TN-5wk), exposed to heat stress for 8 hours (HS-8 h), or exposed to heat stress for 5 weeks (HS-5wk). Left: Heatmap of metabolite abundance. Color intensity reflects standardized metabolite levels (Z-scores). Metabolites that were identified as key regulators in Fig. 3D are annotated in green. Right: 6 distinct metabolite clusters were identified based on similarity in abundance patterns across treatments.

Hierarchical cluster analysis based on metabolite levels across the three experimental conditions identified distinct clusters of metabolites with coordinated responses to heat stress (Fig. 4). Cluster 1, characterized by a gradual increase in abundance from TN-5wk to HS-8 h and HS-5wk, included metabolites involved in amino acid catabolism and nitrogen metabolism, such as creatine, kynurenine, glycerol-3-phosphate, and glutamine. In contrast, cluster 5 consists of metabolites that remained stable after HS-8 h treatment, but decreased in HS-5wk treatments, including metabolites involved in glycolysis (e.g., glucose, lactate) and TCA cycle (e.g., citrate, cis-aconitate), suggesting that long-term heat stress impacts key aspects of energy metabolism. Cluster 6 contained metabolites associated with nucleotide metabolism, including methionine, cytidine, orotate, uridine, and n-carbamoyl-aspartate, all of which were consistently downregulated in both HS-8 h and HS-5wk treatments. The latter metabolites are key intermediates in pyrimidine biosynthesis and methylation reactions, indicating that heat stress may suppress *de novo* nucleotide synthesis or redirect metabolic precursors toward other alternative stress-related processes, independent of the duration of heat stress.

Interestingly, clusters 3 and 4 displayed a distinct pattern in which metabolite levels were increased under short-term heat stress (HS-8 h), but returned to baseline following prolonged exposure to heat (HS-5wk). This pattern was particularly evident for nucleotide-related compounds, highlighting their potential role in the very short-term heat stress response. These included nucleobases (e.g., adenine), nucleosides (e.g., uridine) and nucleotide monophosphates (e.g., AMP).

Taken together, the clustering analysis reinforces and extends the findings from multivariate and univariate findings, demonstrating that heat stress induces coordinated and duration-dependent shifts in metabolic pathways related to nitrogen handling, amino acid metabolism, nucleotide turnover, and energy homeostasis.

### Intermediate and reversible metabolic profiles after heat stress

**Fig. 5:**
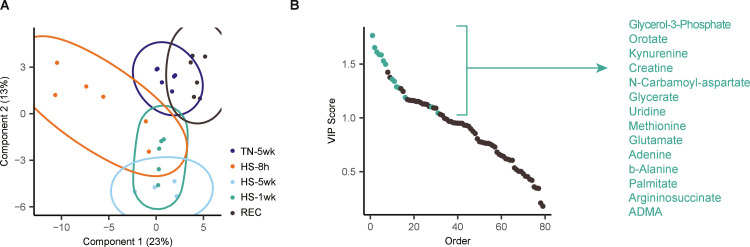
Serum metabolomics analysis of broilers exposed to long-term and very short-term heat stress and recovery. A: Partial least squares-discriminant analysis (PLS-DA) of serum metabolite levels from broilers housed under thermoneutral conditions for 5 weeks (TN-5wk, dark blue), exposed to heat stress for 8 hours (HS-8 h, orange), exposed to heat stress for 1 week (HS-1wk, green), exposed to heat stress for 5 weeks (HS-5wk, light blue) and broilers housed under thermoneutral conditions for 3 weeks, transferred to heat stress conditions for 1 week and back to the thermoneutral conditions for the final week (REC, brown). B: Variable importance in projection (VIP) scores of metabolites ranked by their contribution to group separation. Metabolites with a VIP score > 1 and a statistically significant difference between at least two treatments (*P* < 0.05) are highlighted in green and considered key contributors to the observed metabolic shifts.

Having identified key metabolic alterations associated with short- and long-term heat stress, we next investigated how these profiles evolved following 1 week of heat stress (HS-1wk) and after a recovery period (REC). PLS-DA showed that the metabolic profile of the HS-1wk treatment overlapped with both the HS-8 h and HS-5wk treatment, indicating an intermediate metabolic state (Fig. 5). In contrast, the recovery treatment (REC) clustered closely together with the control treatment (TN-5wk), indicating that the metabolic disturbances induced by heat stress largely normalize after a recovery period of 8 hours.

**Fig. 6:**
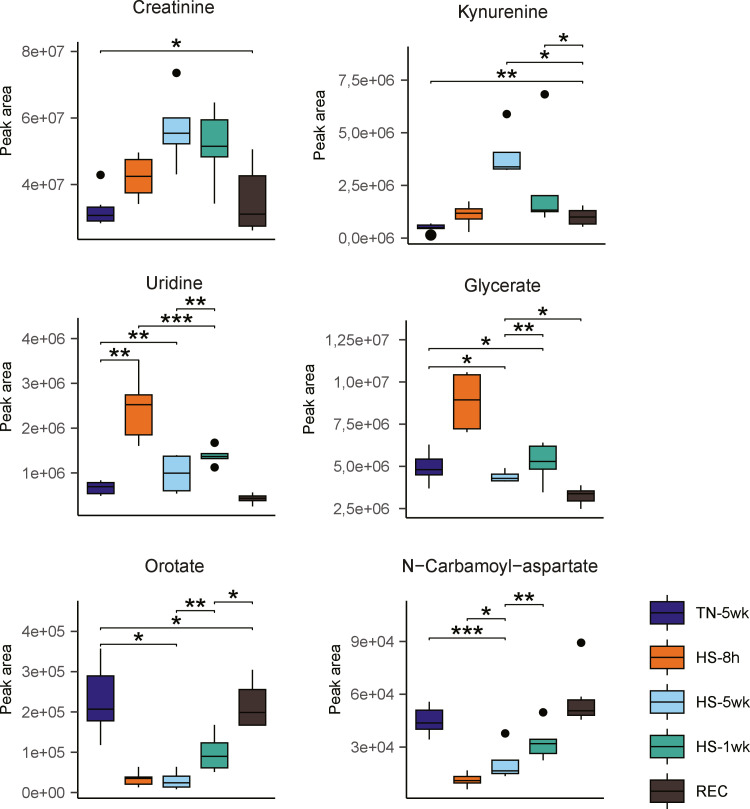
Individual metabolite levels of broilers exposed to long-term and very short-term heat stress and recovery. Boxplots of serum metabolite levels of creatinine, kynurenine, uridine, glycerate, orotate and N-carbamoyl-aspartate from broilers housed under thermoneutral conditions for 5 weeks (TN-5wk, dark blue), exposed to heat stress for 8 hours (HS-8 h, orange), exposed to heat stress for 1 week (HS-1wk, green), exposed to heat stress for 5 weeks (HS-5wk, light blue) and broilers housed under thermoneutral conditions for 3 weeks, transferred to heat stress conditions for 1 week and back to the thermoneutral conditions for the final week (REC, brown). Statistical differences between treatments was assessed using Student’s t-tests with Benjamini-Hochberg post hoc correction (* = *P<* 0.05, ** = *P<* 0.01, *** = *P<* 0.001).

At the level of individual metabolites, several patterns confirmed the finding of the PLS-DA (Fig. 6). Creatinine and kynurenine, which were increased in the HS-5wk treatment, returned to control levels in the REC treatment. Orotate and n-carbamoyl-aspartate, which were consistently decreased in both HS-8 h and HS-5wk treatments, also returned to control levels in the REC treatment. In the HS-1wk treatment, the levels of these metabolites were partially restored compared to the heat stress treatments, but not yet at baseline. Uridine and glycerate, identified as markers for HS-8 h, similarly returned to control levels in the recovery treatment and showed transient elevation in the HS-1wk treatment. Together, these findings indicate that the metabolic effects of heat stress are at least partially reversible, with most biomarkers returning to baseline after recovery. The complete hierarchical clustering including the HS-1wk and REC treatments can be found in Supplemental Fig. 1.

## Discussion

In this study, cyclic heat stress was effectively induced by natural high ambient temperatures characteristic of the region's tropical climate. The resulting increase in cloacal temperature underlined the broilers' inability to adequately thermoregulate, thereby confirming a physiological state of heat stress. This rise in cloaca temperature was accompanied by a marked decline in growth performance and feed efficiency, as has been previously reported by Liu et al. (2020). By applying LC-MS-based metabolomics, we demonstrate that the duration of heat stress exposure plays a key role in shaping the metabolic response. Our findings clearly distinguish the metabolic signatures of very short-term (8 h) and long-term (5 weeks) heat stress. While the response to very short-term heat stress was uniquely characterized by an increase in metabolites involved in nucleotide metabolism, such as uridine, long-term heat stress induced a catabolic metabolic phenotype, evidenced by elevated plasma levels of kynurenine, creatinine, and argininosuccinate. On the other hand, reduced levels of methionine, cytidine, orotate and n-carbamoyl-aspartate was independent of the duration of HS, as both very short-term and long-term HS affected these metabolites. These differences suggest that heat exposure triggers transient metabolic adjustments to cope with heat stress for a shorter time, while longer exposure to heat elicits a lasting response involving muscle catabolism and amino acid degradation.

The increased plasma uridine levels observed after 8 hours of heat exposure point to activation of the pyrimidine salvage pathway. Under heat stress, increased production of reactive oxygen species (ROS) has been shown to induce cellular damage, resulting in enhanced DNA damage and nucleotide turnover (Shimamoto et al. (2020). Uridine not only serves as a precursor for nucleotide salvage, but has also been reported to have antioxidative activity (Song et al., 2025). Therefore, the elevated plasma uridine observed in our 8-hour HS group can reflect an acute, systemic protective response simultaneously providing direct antioxidant defence and maintaining nucleotide availability for DNA repair via the salvage pathway. The rapid return to control levels after recovery or prolonged heat stress suggests that these changes are reversible and highlight uridine as a potential sensitive marker for acute heat stress.

The decreased levels of orotate and n-carbamoyl-aspartate after short and long durations of heat stress indicate reduced de novo nucleotide biosynthesis potentially shifting the balance towards nucleotide salvage to maintain nucleotide availability. This metabolic shift supports immediate cellular demands for RNA turnover, DNA repair or immune-related activity during early heat stress response. These results are of interest when taken together with results from duodenal samples from heat stressed broilers, in which nucleotide salvage was suggested (Dridi et al., 2022). When integrating the local tissue-level findings with our plasma metabolomic profile, it is evident that this downregulation of *de novo* pyrimidine synthesis represents a system-wide adaptation, in order to conserve energy without compromising DNA repair.

Long-term exposure to heat (5 weeks) elicited a pattern consistent with catabolic activity, reflected by elevated levels of creatinine, kynurenine and arginosuccinate. Creatinine and argininosuccinate are linked to muscle- and protein breakdown, while kynurenine is a marker of tryptophan catabolism. Together, these changes imply increased proteolysis, amino acid degradation, possibly as a strategy to support energy production and maintain homeostasis under prolonged thermal challenge. These changes are in line with previous studies showing reduced muscle accretion, increased oxidative stress, and altered nitrogen metabolism in chronically heat-stressed broilers (de Souza et al., 2016; Tomonaga et al., 2018). As reviewed by Belhadj Slimen et al. (2016), reorganization of post-absorptive metabolism is a highly conserved adaptive response; under prolonged thermal challenge, the physiological priority shifts away from growth and muscle accretion, and moves towards catabolism.

In addition, lower levels of glucose, lactate and TCA cycle intermediates, indicative of lower glycolytic and mitochondrial activity. Although reduced metabolite levels may reflect either decreased production or increased utilization, these findings are in line with Li et al. (2024), who reported reduced mitochondrial energy metabolism and increased lipid synthesis in heat stressed broiler muscle. A potential mechanistic explanation is impaired TCA cycle flux, possibly linked to the accumulation of inhibitory metabolites such as malonate (Zampiga et al. (2021). Increased lipid deposition during heat stress appears paradoxical given that fat oxidation has been proposed to be favoured under heat stress due to its lower heat increment (Teyssier et al., 2022).

Interestingly, glycerol-3-phosphate levels were increased under chronic heat stress. Glycerol-3-phosphate has a central position between glucose metabolism and lipid metabolism, and plays a role in both triglyceride synthesis and redox balance via the glycerol-3-phosphate shuttle. Its accumulation may therefore reflect two complementary processes: a redirection of glycolytic intermediates toward lipid biosynthesis due to reduced TCA cycle flux, or a disruption of redox balance resulting from impaired mitochondrial oxidation of NADH.

In addition to distinguishing the metabolic profiles associated with different durations of heat stress, this study shows that these metabolic alterations are largely reversible. Broilers returning to thermoneutral conditions following 1 week of heat exposure exhibited metabolic profiles resembling unstressed controls. HS-1wk broilers displayed intermediate patterns, consistent with an adaptation phase, while recovery was nearly complete within 8 hours. These results underscore that the physiological response to heat stress is a highly dynamic process and highlight the capacity of broilers to recover metabolically from heat stress.

From a practical perspective, the identification of metabolites with heat-stress specific patterns opens new opportunities for the development of biomarkers for monitoring broiler health under heat stress. There is a strong need for an objective biomarker to detect heat stress in poultry. Human observation is most of the time too subjective and can be biased in either direction. Compounds such as uridine and kynurenine may be useful as early indicators of acute or chronic heat stress. However, these metabolites are also involved in other stress related reactions, such as immune activation and oxidative stress responses. As such, their specificity as a marker for heat stress must be further validated across different types of stressors and physiological states, before implementation.

We focused on a defined set of polar metabolites and while these metabolites are part of essential metabolic pathways including the TCA cycle, glycolysis, amino acid metabolism and nucleotide synthesis, a large part of the metabolome is not covered, particularly lipids. Lipid metabolism is linked to thermoregulation, oxidative stress, inflammation and energy metabolism. Lipidomics analysis may therefore reveal additional candidate biomarkers. Future studies should quantify a broader set of metabolites in order to provide a complete picture of the metabolome of the heat stressed broilers.

It is important to consider that the negative effects of HS are often presented in a confounded way. It can be argued that some effects cannot be directly attributed to HS but to a secondary response, such as reduced feed intake or increased water intake. It is therefore crucial to distinguish between these effects by means of an isoenergetic feed regime with and without the presence of a thermal challenge. Furthermore, future studies should incorporate more sampling moments in a 24 h period, therefore identifying whether the systemic metabolome of a heat stressed broiler transiently returns to baseline during the cooler phases of a cyclic heat stress model.

In conclusion, metabolomics can be used as a tool to identify responses caused by heat stress and also differentiate between short-term and long-term exposure. Secondly, broilers are resilient to HS, as they can return to metabolic profiles typical of thermoneutral conditions within a very short period of time. Uridine and kynurenine were identified as promising biomarkers for distinguishing acute from chronic thermal load, their specificity warrants further validation.

## CRediT authorship contribution statement

**A. Papanikolaou:** Writing – original draft, Methodology, Investigation, Formal analysis, Conceptualization. **E.M.J.M. Kampschoer:** Writing – original draft, Investigation, Formal analysis, Conceptualization. **M.G.R. Matthijs:** Writing – review & editing, Supervision, Project administration, Funding acquisition. **J.T. Schonewille:** Writing – review & editing, Supervision, Funding acquisition, Conceptualization. **S. Khempaka:** Supervision, Resources, Project administration. **C.R. Berkers:** Writing – review & editing, Supervision, Methodology. **E.A. Zaal:** Writing – review & editing, Writing – original draft, Supervision, Methodology, Formal analysis, Data curation.

## Disclosures

The authors declare that they have no conflicts of interest to report.

## Appendix Supplementary materials

Supplemental Fig. S1. Hierarchal clustering of serum metabolites and cluster-specific metabolic profiles in broilers under heat stress. Hierarchical clustering analysis was performed on all detected serum metabolites from broilers housed under thermoneutral conditions for 5 weeks (TN-5wk), exposed to heat stress for 8 hours (HS-8 h), exposed to heat stress for 1 week (HS-1wk), exposed to heat stress for 5 weeks (HS-5wk) and the recovery group (REC). Left: Heatmap of metabolite abundance. Color intensity reflects standardized metabolite levels (Z-scores). Metabolites that were identified as key regulators in Fig. 3D are annotated in green. Right: 6 distinct metabolite clusters were identified based on similarity in abundance patterns across treatments. Image, application 1

## Footnotes

Supplementary material associated with this article can be found, in the online version, at doi:10.1016/j.psj.2026.106876. Supplemental Fig. S1. Hierarchal clustering of serum metabolites and cluster-specific metabolic profiles in broilers under heat stress. Hierarchical clustering analysis was performed on all detected serum metabolites from broilers housed under thermoneutral conditions for 5 weeks (TN-5wk), exposed to heat stress for 8 hours (HS-8 h), exposed to heat stress for 1 week (HS-1wk), exposed to heat stress for 5 weeks (HS-5wk) and the recovery group (REC). Left: Heatmap of metabolite abundance. Color intensity reflects standardized metabolite levels (Z-scores). Metabolites that were identified as key regulators in Fig. 3D are annotated in green. Right: 6 distinct metabolite clusters were identified based on similarity in abundance patterns across treatments.

## References

[bib0001] Belhadj Slimen I., Najar T., Ghram A., Abdrrabba M. (2016). Heat stress effects on livestock: molecular, cellular and metabolic aspects, a review. J. Anim. Physiol. Anim. Nutr..

[bib18] Cobb-Vantress (2021).

[bib0002] Cui Y., Wang C., Hao Y., Gu X., Wang H. (2019). Chronic heat stress induces acute phase responses and serum metabolome changes in finishing pigs. Animals.

[bib0003] de Souza L.F.A., Espinha L.P., de Almeida E.A., Lunedo R., Furlan R.L., Macari M. (2016). How heat stress (continuous or cyclical) interferes with nutrient digestibility, energy and nitrogen balances and performance in broilers. Livest. Sci..

[bib0004] Dridi J.S., Greene E..S., Maynard C.W., Brugaletta G., Ramser A., Christopher C.J., Campagna S.R., Castro H.F., Dridi S. (2022). Duodenal metabolic profile changes in heat-stressed broilers. Animals.

[bib0005] Li X., Zhao X., Yu M., Zhang M., Feng J. (2024). Effects of heat stress on breast muscle metabolomics and lipid metabolism related genes in growing broilers. Animals.

[bib0006] Liu L., Ren M., Ren K., Jin Y., Yan M. (2020). Heat stress impacts on broiler performance: a systematic review and meta-analysis. Poult. Sci..

[bib0007] Lu Z., He X., Ma B., Zhang L., Li J., Jiang Y., Zhou G., Gao F. (2018). Serum metabolomics study of nutrient metabolic variations in chronic heat-stressed broilers. Br. J. Nutr..

[bib0008] Mangan M., Siwek M. (2024). Strategies to combat heat stress in poultry production—a review. J Anim Physiol Anim Nutr.

[bib17] National Research Council, & Subcommittee on Poultry Nutrition (1994).

[bib0009] Nawab A., Ibtisham F., Li G., Kieser B., Wu J., Liu W., Zhao Y., Nawab Y., Li K., Xiao M. (2018). Heat stress in poultry production: mitigation strategies to overcome the future challenges facing the global poultry industry. J. Therm. Biol..

[bib0011] Pang Z., Lu Y., Zhou G., Hui F., Xu L., Viau C., Spigelman A.F., MacDonald P.E., Wishart D.S., Li S. (2024). MetaboAnalyst 6.0: towards a unified platform for metabolomics data processing, analysis and interpretation. Nucleic. Acids. Res..

[bib0012] Shimamoto S., Nakamura K., Tomonaga S., Furukawa S., Ohtsuka A., Ijiri D. (2020). Effects of cyclic high ambient temperature and dietary supplementation of orotic acid, a pyrimidine precursor, on plasma and muscle metabolites in broiler chickens. Metabolites.

[bib0013] Song C., Liu Z.J., Xu B., Zou R., Hu W. (2025). The role of uridine in health and disease. J. Inflamm. Res..

[bib0014] Teyssier J., Preynat A., Cozannet P., Briens M., Mauromoustakos A., Greene E., Owens C., Dridi S., Rochell S. (2022). Constant and cyclic chronic heat stress models differentially influence growth performance, carcass traits and meat quality of broilers. Poult. Sci..

[bib0015] Tomonaga S., Okuyama H., Tachibana T., Makino R. (2018). Effects of high ambient temperature on plasma metabolomic profiles in chicks. Anim. Sci. J..

[bib0010] Xie J., Tang L., Lu L., Zhang L., Lin X., Liu H.C., Luo X. (2015). Effects of acute and chronic heat stress on plasma metabolites, hormones and oxidant status in restrictedly fed broiler breeders. Poult. Sci..

[bib0016] Zampiga M., Laghi L., Zhu C., Mancinelli A.C., Mattioli S., Sirri F. (2021). Breast muscle and plasma metabolomics profile of broiler chickens exposed to chronic heat stress conditions. Animal.

