# Supplementary figures and images for: Plasma metabolomics reveals distinct responses to acute and chronic heat stress in broilers
Source: Poult Sci. 2026 Mar 28;105(7):106876. doi: 10.1016/j.psj.2026.106876 (PMC13087746; doi:10.1016/j.psj.2026.106876)

Supplemental Figure S1

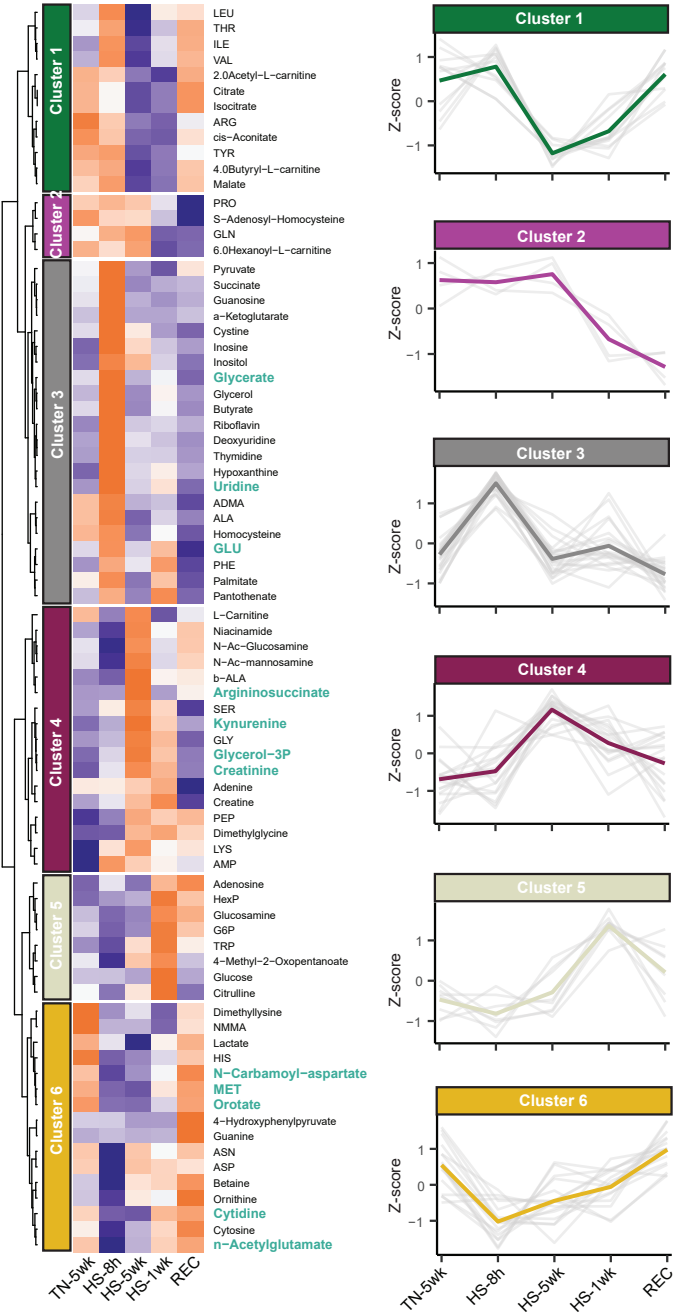

Supplement: Supplementary file 1 — Supplemental Fig. S1. Hierarchal clustering of serum metabolites and cluster-specific metabolic profiles in broilers under heat stress. Hierarchical clustering analysis was performed on all detected serum metabolites from broilers housed under thermoneutral conditions for 5 weeks (TN-5wk), exposed to heat stress for 8 hours (HS-8 h), exposed to heat stress for 1 week (HS-1wk), exposed to heat stress for 5 weeks (HS-5wk) and the recovery group (REC). Left: Heatmap of metabolite abundance. Color intensity reflects standardized metabolite levels (Z-scores). Metabolites that were identified as key regulators in Fig. 3D are annotated in green. Right: 6 distinct metabolite clusters were identified based on similarity in abundance patterns across treatments. [file mmc1.pdf]
